# Supplementary material for: Crawling and Gliding: A Computational Model for Shape-Driven Cell Migration
Source: PLoS Comput Biol. 2015 Oct 21;11(10):e1004280. doi: 10.1371/journal.pcbi.1004280 (PMC4619082; doi:10.1371/journal.pcbi.1004280)
Supplement: S1 Code — (ZIP) [file pcbi.1004280.s012.zip › release/tst/doc/html/dish_8h.html]

Tissue Simulation Toolkit: dish.h File Reference


Tissue Simulation Toolkit
 0.1.4.1


- Main Page
- Namespaces
- Classes
- Files

- File List
- File Members

Classes |
Namespaces |
Macros |
Enumerations

dish.h File Reference

`#include <vector>`  
`#include "graph.h"`  
`#include "random.h"`  
`#include "pde.h"`  
`#include "cell.h"`  
`#include "ca.h"`

Include dependency graph for dish.h:

This graph shows which files directly or indirectly include this file:

Go to the source code of this file.

|  |  |
| --- | --- |
| Classes | |
| class | Dish |
|  | The virtual Petri dish. More... |
|  | |

|  |  |
| --- | --- |
| Namespaces | |
|  | ColourMode |
|  | |

|  |  |
| --- | --- |
| Macros | |
| #define | INIT   void Dish::Init(void) |
|  | |

|  |  |
| --- | --- |
| Enumerations | |
| enum | { ColourMode::State, ColourMode::CellType, ColourMode::Sigma, ColourMode::Auxilliary } |
|  | |

## Macro Definition Documentation

#define INIT   void Dish::Init(void)


---

Generated on Thu Aug 14 2014 22:04:01 for Tissue Simulation Toolkit by  

 1.8.6
